# The individual role of peanut proteins Ara h1, 2, 3 and 6 in peanut allergy

**DOI:** 10.1186/2045-7022-1-S1-O36

**Published:** 2011-08-12

**Authors:** Smit Joost, Pennings Maarten, Houben Geert, Els van Hoffen, Pieters Raymond

**Affiliations:** 1 Institute for Risk Assessment Sciences, Utrecht University, Utrecht, Netherlands; 2 Utrecht University Medical Center, Utrecht, Netherlands; 3 TNO, Zeist, Netherlands

## 

One of the major allergenic food or food constituent is peanut, in which a number of allergenic proteins have been described (Ara h1-9). Until now, the relative contribution of the individual peanut allergens to clinical food allergic responses is not known. Therefore, the present studies aimed to elucidate the relative contribution of Ara h1, 2, 3, and 6 to peanut allergy in a mouse model.

For this purpose, mice were immunized by oral gavage with a whole peanut protein extract or with purified allergens Ara h1, 2, 3, or 6. Hereafter, mice were challenged with the individual allergens and blood was collected to measure allergen-specific antibodies and mast cell degranulation (MMCP-1 in serum). Spleens were harvested to measure allergen-specific T-cell reactivity. To assess the potential of the individual peanut proteins to induce anaphylaxis, mice were sensitised with whole peanut protein extract and challenged intraperitoneally with purified Ara h1, 2, 3 or 6.

Sensitisation with whole peanut extract induced Ara h1, 2, 3 and 6 specific IgE, IgG1 and IgG2a. In addition, sensitisation with the individual peanut allergens elicited antibody responses with specificity to the allergen used. T cell cultures showed Th1 and Th2 type cytokine production upon restimulation with both peanut extract and the individual peanut allergens. Interestingly, only Ara h2 and 6 were able to elicit mast cell degranulation after oral challenge. In contrast, after systemic challenge, Ara h1 and 2 were able to elicit strong anaphylactic responses, whereas anaphylactic responses induced by Ara h3 and 6 were less severe. In conclusion, individual peanut allergens do not differ drastically in the capacity to sensitize via the oral route. Interestingly, depending on the route of provocation, peanut proteins differ in their capacity to cause effector responses such as mast cell degranulation and anaphylaxis. In future studies, the mechanism behind the functional differences of individual peanut allergens and their cross-reactivity on T cell and antibody level will be investigated.

